# Neutron and X-ray Diffraction Analysis of Macro and Phase-Specific Micro Residual Stresses in Deep Rolled Duplex Stainless Steels

**DOI:** 10.3390/ma14081854

**Published:** 2021-04-08

**Authors:** Samuel Pulvermacher, Tobias Bücker, Jan Šaroun, Joana Rebelo-Kornmeier, Michael Hofmann, Jens Gibmeier

**Affiliations:** 1 Institute for Applied Materials, Karlsruher Institut für Technologie, Kaiserstraße 12, 76131 Karlsruhe, Germany; samuel.pulvermacher@kit.edu (S.P.); tobias.buecker@student.kit.edu (T.B.); 2 Nuclear Physics Institute of the ASCR, 250 68 Řež, Czech Republic; saroun@ujf.cas.cz; 3 Heinz Maier-Leibnitz Zentrum (MLZ), TU München, D-85748 Garching, Germany; joana.kornmeier@frm2.tum.de (J.R.-K.); michael.hofmann@frm2.tum.de (M.H.)

**Keywords:** residual stress measurement, phase specific stress, macro stress, phase-specific micro residual stresses, duplex steel, neutron diffraction, surface effect, phase specific texture, soft X-ray *sin*^2^*ψ*, incremental hole drilling

## Abstract

Experimental analyses of depth distributions of phase-specific residual stresses after deep rolling were carried out by means of laboratory X-ray diffraction and neutron diffraction for the two duplex steels X2CrNiMoN22-5-3 and X3CrNiMoN27-5-2, which differ significantly in their ferrite to austenite ratios. The aim of the investigation was to elucidate to which extent comparable results can be achieved with the destructive and the non-destructive approach and how the process induced phase-specific micro residual stresses influence the determination of the phase- and {*hkl*}-specific reference value d_0_, required for evaluation of neutron strain scanning experiments. A further focus of the work was the applicability of correction approaches that were developed originally for single-phase materials for accounting for spurious strains during through surface neutron scanning experiments on coarse two-phase materials. The depth distributions of macro residual stresses were separated from the phase-specific micro residual stresses. In this regard, complementary residual stress analysis was carried out by means of incremental hole drilling. The results indicate that meaningful macro residual stress depth distributions can be determined non-destructively by means of neutron diffraction for depths starting at about 150–200 µm. Furthermore, it was shown that the correction of the instrumental surface effects, which are intrinsic for surface neutron strain scanning, through neutron ray-tracing simulation is applicable to multiphase materials and yields reliable results. However, phase-specific micro residual stresses determined by means of neutron diffraction show significant deviations to data determined by means of lab X-ray stress analysis according to the well-known *sin*^2^*ψ*-method.

## 1. Introduction

Duplex stainless steels combine properties of austenitic and ferritic stainless steels, i.e., they generally have good mechanical properties, including high strength and ductility, and corrosion resistance is often better than in conventional austenitic steels [1,2,3]. Due to the high resistance to corrosion, duplex stainless steels are mainly used in the chemical industry, petrochemical industry, seawater desalination plants and in offshore technology [4,5]. The condition of the outermost surface layers is of utmost importance for the functionality and service life of highly stressed structural and functional components. Many degradation processes such as corrosion or the nucleation of fatigue cracks have their origin at the surface. Hence, surface layer modification is a central step in the production of many high-performance components. Mechanical surface treatments like shot peening, laser shock peening, deep rolling or hammering are well-established post treatment processes [6,7] that are often applied to enhance the load bearing capacity of technical components. By these means, the near surface region is work hardened and therefore compressive residual stresses (RS) are induced. These compressive RS can impede crack initiation and propagation; hence, in most cases they are regarded as beneficial for technical applications [7,8,9]. However, for coarse multiphase materials such as duplex stainless steels, the appropriate assessment of RS induced by means of post treatments requires the separation of micro and macro RS, since, in these cases, the material’s load bearing capacity strongly depends on the load partitioning on the phases under applied load and on the phase-specific mechanical behaviour [10,11,12,13,14]. Hence, for the assessment of the stress susceptibility of duplex stainless steels, knowledge on the phase-specific RS induced by the manufacturing or post treatment is essential. This can only be provided through diffraction-based RS analysis methods [15].

The crux for RS analysis on duplex stainless steels is that often phase-specific crystallographic textures exist that complicate experimental stress determination as well as the appropriate evaluation of the measured data [10,11].

Here, we aim at the assessment of the suitability of neutron diffraction for non-destructive analysis of phase-specific RS depth distributions that are induced by defined deep rolling treatments for commonly applied duplex stainless steels. In this methodical approach, two duplex steels were chosen that clearly differ in their phase fractions of ferrite (α-Fe) and austenite (γ-Fe). As model materials, the widely used duplex steels X2CrNiMoN22-5-3 (1.4462), with a nominal ferrite to austenite volume ratio of 50:50, and X3CrNiMoN27-5-2 (1.4460), with a ferrite to austenite volume ratio of 70:30, were chosen. The range of the near surface region typically affected by the deep rolling process is around one millimetre. Hence, regarding neutron diffraction stress analysis, through surface strain scanning must be carried out, i.e., the sample surface must be scanned stepwise through the nominal gauge volume, which is determined by the optics used in the primary and the secondary neutron beam paths (slit systems, radial collimators and parabolic guides). By these means, intrinsic difficulties arise which are related to the fact that by through surface strain scanning in the first steps, the nominal gauge volume is only partially immerged in the sample surface. A nominal gauge volume that is only partially filled with material causes the so called “surface effect” [16,17,18], which furthermore results in spurious strains. For meaningful RS evaluations, these spurious strains must be taken into consideration and either must be minimized through appropriate measuring strategies or must be corrected, for example, by suitable simulations of the diffraction experiment. For single-phase materials, this correction has been successfully applied using the freely available simulation software “SIMRES” [19,20,21]. However, in case of coarse multiphase materials, the suitability of this correction approach must be examined and assessed. Therefore, regarding the assessment of the neutron diffraction approach determining near surface RS depth distributions induced by deep rolling of duplex stainless steel, supplementary metallographic analyses of the microstructure and phase-specific texture analysis were performed. Texture analysis was carried out in the initial state and after deep rolling. Here, the texture evolution for different depth was considered. Furthermore, phase-specific instrumented hardness testing was carried out to assess which of the phases exhibits the higher work hardening effect as a consequence of the mechanical surface treatment. Our neutronographic RS analysis was further assessed by means of complementary approaches, which are destructive or at least semi-destructive. In addition to neutron diffraction experiments carried out at the STRESS-SPEC instrument [22] at the MLZ research reactor FRM II in Garching, Germany, phase-specific RS analysis with the well-established *sin*^2^*ψ*-method [15] was performed using conventionally generated soft lab X-rays. Here, for the determination of RS depth gradients, a successive layer removal must be carried out, for instance, by means of electrochemical polishing, in combination with the reapplication of the *sin*^2^*ψ*-analysis on the newly generated surfaces. Finally, the macro RS that are induced by deep rolling are determined by means of the incremental hole drilling method [23].

## 2. Materials and Deep Rolling Procedure

The two widely used duplex stainless steel alloys X2CrNiMoN22-5-3 (1.4462) and X3CrNiMoN27-5-2 (1.4460) were chosen for the investigations. Table 1 shows the nominal chemical composition. 

The delivery state of duplex steel 1.4462 was a hot rolled plate with a sheet thickness of 10 mm. Duplex steel 1.4060 was delivered as hot rolled rod material of ø 25 mm. From this delivery state, plate like samples were manufactured with dimension of 40 × 25 × 10 mm^3^ and of 40 × ø 25 × 10 mm^3^, respectively. 

Table 2 shows the nominal phase contents as well as the nominal strength values for both materials in comparison to measured data. The strength values were determined by means of uniaxial tensile tests. The average phase fractions were determined by means of X-ray diffraction phase analysis. Here, the 6-line method [26] was applied using MoKα-radiation.

**Figure 1:**
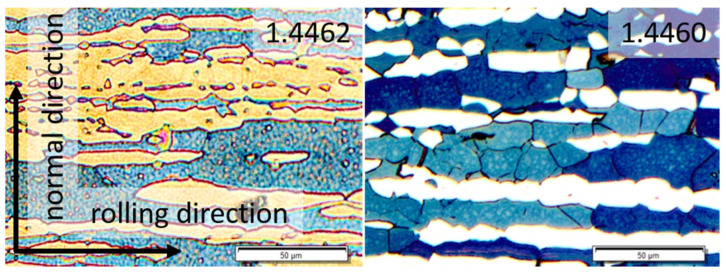
Cross sectional views of the microstructure of duplex steels X2CrNiMoN22-5-3 (1.4462) and X3CrNiMoN27-5-2 (1.4460) in the initial state (in the centre of the plate). Etchant: Beraha II; bright regions: austenite; dark regions: ferrite. The directions of the plate coincides with the subsequent deep rolling directions.

Figure 1 shows cross sections of the microstructures of the two duplex steels. On basis of the metallographic investigations, the mean grain sizes were determined.

**Figure 2:**
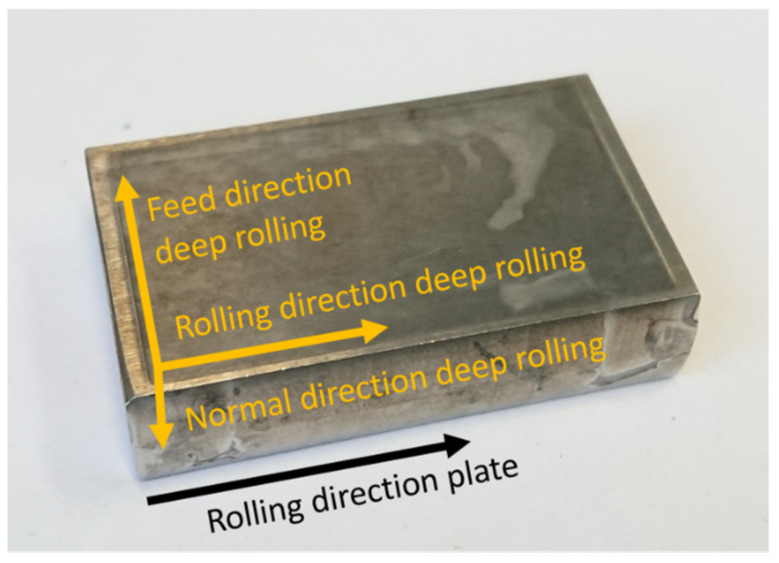
Sample geometry and assignment of the sample coordinate system.

For duplex steel X2CrNiMoN22-5-3 (1.4462), the metallographically determined average grain sizes, after deep rolling, were about 20.9 μm in the rolling direction (RD), about 14.9 μm in the feed direction (FD) and about 6.7 μm in the normal direction. The sample coordinate system used is shown in Figure 2. For the duplex steel X3CrNiMoN27-5-2 (1.4460), the grain sizes, after deep rolling, were about 15.3 μm in the rolling direction (RD), about 11.1 μm in the feed direction and approx. 11 μm in the normal direction, respectively. Prior to deep rolling, the macro RS were determined by means of incremental hole drilling. Technically, the samples can be considered stress free with macro RS of less than 50 MPa.

**Figure 3:**
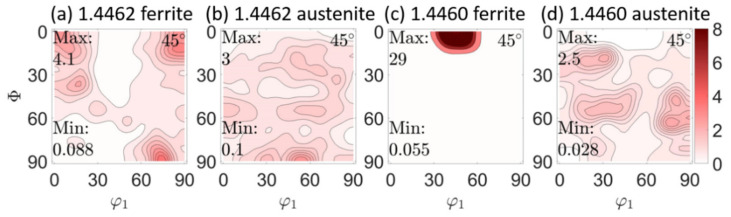
*φ*_2_ = 45°—projections through the orientation distribution functions (ODF) in the initial states of both duplex stainless steels. (**a**,**b**) for steel 1.4462 for the ferrite and the austenite phase, respectively and (**c**,**d**) for steel type 1.4460 for the ferrite and the austenite phase, respectively.

In the initial state, the phase-specific crystallographic textures were determined. Figure 3 shows that the *φ*_2_ = 45°—projections through the orientation distribution functions (ODF) that were determined by means of X-ray diffraction using CoKα-radiation.

Deep rolling was carried out using a deep rolling tool of type HG6 from Ecoroll (Celle Germany), with a hydrostatically supported ball with a diameter of 6 mm using, as process parameters, a pressure of 260 bars, a path distance of 0.03 mm and a feed rate of 2000 mm/min. 

## 3. Experimental Procedures

### 3.1. Neutron Diffraction

The neutronographic RS analyses were performed at the STRESS-SPEC instrument [22] at the neutron research reactor FRM II (Garching Germany). A wavelength of 1.67 Å was defined through the Si-monochromator using a take-off angle of 75.9°. For austenite, the lattice planes {311} were measured, and, for ferrite, the {211} lattice planes were considered. These two planes were chosen in accordance with the literature because of the low anisotropy effect and should, therefore, generally allow a reliable RS analysis [27]. The individual diffraction lines were fitted using Gaussian functions by means of the software package STeCa^2^ [28]. The spurious strains that occur as an effect of the partially filled gauge volume at the through surface strain scanning were corrected numerically by use of the simulation tool SIMRES [20,21]. For the simulations, the applied measuring parameters were used as input parameters. On the primary beam path, a slit with a nominal width of 1 mm was used in combination with a radial collimator with a full width at half maximum of FWHM = 1 mm. Hence, the nominal gauge volume was about 1 × 1 × 10 mm^3^, and, for strain scanning of the reference pins, a 1 × 1 × 1 mm^3^ gauge volume was chosen. A very small step size of 0.1 mm was selected for immersing the bulk sample/pin into the nominal gauge volume. After coordinate matching [18], the lattice strain values of the bulk sample and the pin, which form a pair, i.e., corresponding to the same coordinate, were calculated with each other.

For strain calculation, the interplanar spacing of the stress-free lattice *d*_0_ must be known as the reference value. The lattice strain is defined by [15]:

(1) $$\varepsilon^{hkl}=\frac{d^{hkl}-d_{0}^{hkl}}{d_{0}^{hkl}}$$

**Figure 4:**
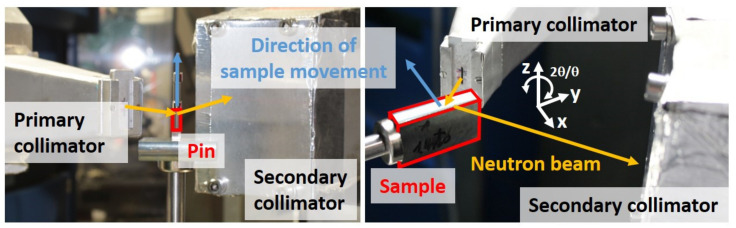
Principle measurement setup. Left: Z-mode for reference sample measurement. Right: Measurement of the bulk sample in the rolling direction.

The reference parameter *d*_0_ was determined from a small pin with a diameter of about ø 1.7 mm that was sectioned from a deep rolled plate by means of wire electro discharge machining (EDM). The measurement of the reference pin was performed in z-scan mode (see Figure 4), i.e., through surface scanning was carried out with the scan axis being parallel to the goniometer axis of the 2θ/θ-goniometer of the STRESS-SPEC set-up. By these means, only a correction for the measurement center of gravity is required, since, with this kind of scan, no surface effects occur and no spurious strains, due to partially filled nominal gauge volumes, must be considered and corrected. For the pin, only the rolling (RD) and the feed direction (FD) were measured. For strain calculation in the normal direction, a constant value from the base material, i.e., outside the region affected by the deep rolling, was applied.

For residual stress analysis, we assumed that the principal directions correspond with the rolling direction (RD), the feed direction (FD) and the direction normal to the deep rolled plates (ND) (see Figure 2). Hence, lattice strains were determined in these three principal directions, and, with the lattice strains in the principal directions, the RS components can be calculated according to [18] by:

(2) $$\sigma_{ij}^{hkl}=\frac{E^{hkl}}{\left(1-\upsilon^{hkl}\right)}\left[\varepsilon_{ij}^{hkl}+\frac{\upsilon^{hkl}}{\left(1-2\upsilon^{hkl}\right)}*\left(\varepsilon_{11}^{hkl}+\varepsilon_{22}^{hkl}+\varepsilon_{33}^{hkl}\right)\right]$$

with *i, j* = 11 for RD, 22 for FD and 33 for ND and $E^{hkl\;}$= {*hkl*}-dependent Young’s Modulus and $\upsilon^{hkl}\;$= {*hkl*}-dependent Poisson’s ratio. Throughout the entire paper, the {*hkl*} dependent diffraction elastic constants (DEC) are used as given in Table 3. The austenite values are based on single-crystal elastic constants by [29] and the ferrite values on data from [30]. The plates were measured in all three directions. The chosen measurement mode was transmission in the rolling direction, and reflection in the feed direction transmission and in the normal direction.

### 3.2. X-ray Diffraction

Complimentary to the neutron diffraction stress analyses, X-ray RS analyses according to the *sin*^2^*ψ*-method [15] were carried out. For these analyses, vanadium filtered CrKα-radiation was applied and a 3-circle diffractometer in ψ-geometry was used. As primary aperture, a pinhole collimator with a diameter of ø 2 mm was utilized. On the secondary side, a 4 mm slit was installed in front of the scintillation counter. For the ferrite phase, the {211} lattice planes were chosen, and, for austenite, the {220} lattice planes were considered. A total of 21 sample tilts, in the range of −60° ≤ ψ ≤ 60° equidistantly distributed in *sin*^2^*ψ*, were used. After background subtraction, the interference lines were fitted using Pearson VII functions. K_α__2_ stripping was performed by means of a double peak fitting. For stress calculation, the diffraction elastic constants, as listed in Table 3, were chosen. 

In addition to X-ray stress analyses, an X-ray texture analysis was carried out. These measurements were performed on a Seifert PTS 3000 4-circle-diffractometer using CoKα-radiation. Here, a pinhole collimator of ø 1 mm was used in combination with a 4 mm slit on the secondary side. For the ferrite phases, the three independent lattice planes, {200}, {211} and {220}, were considered, and, for the austenite phase, the {220}, {222} and {311} planes were chosen. For both phases, the orientation distribution function was calculated using the MATLAB software MTEX [31].

### 3.3. Incremental Hole Drilling Method

The Incremental hole drilling technique was applied using a hole drilling device of type RS100, manufactured by Vishay Measurements Group, equipped with a pneumatic high-speed turbine. A blind hole of approx. 1.7 mm in diameter was introduced stepwise through a six-bladed (TiN-coated) tungsten carbide end mill of 1.6 mm nominal diameter from Komet, Brasseler GmbH & Co. KG (Lemgo, Germany). Strain relaxations were recorded using strain gage rosettes of type CEA-06-062UM-120 from Vishay Measurements Group GmbH (type B according to [32], Heilbronn, Germany and a carrier frequency amplifier of type Picas from Peekel Instruments GmbH (Bochum, Germany). For calculation of the RS depth distribution, the differential approach was applied, as proposed by [23], using the elastic constants E = 200 GPa [24,25] and ν = 0.3 for both duplex steels.

### 3.4. Microhardness Testing 

The phase-specific microhardness depth distributions were recorded by means of instrumented hardness testing according to ISO 14577 [33,34,35,36]. Martens hardness (HM) was determined for both phases, ferrite and austenite, on carefully metallographically prepared samples, using a microhardness testing system of type Fischerscope H100 from Fischer, (Sindelfingen, Germany), with a test load of 40 mN, and a Vickerspyramid as indentor.

## 4. Results and Discussion

### 4.1. Phase-Specific Work Hardening

**Figure 5:**
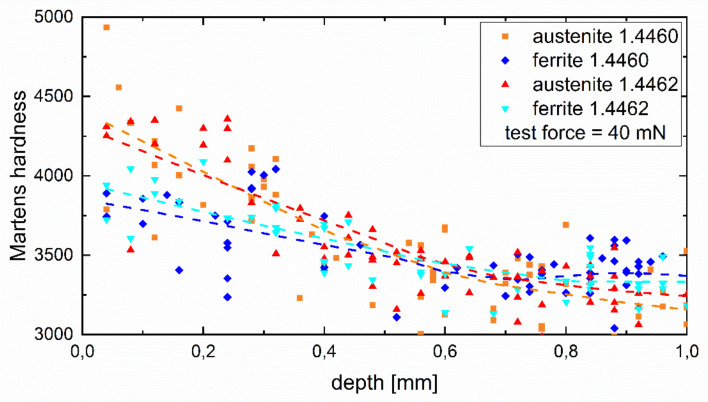
Phase-specific micro hardness of both investigated duplex steels.

Figure 5 shows the results of instrumented hardness testing carried out on selected grains of the two phases, ferrite and austenite, for both duplex steels. The data show a rather large scatter that is typical for this kind of analysis and which can be explained by the rather limited statistics, not least because the assessment of whether the individual hardness value can be clearly assigned to one of the phases is based on the planar (2D) cut. However, apart from this limitation, the levelling curves, based on regression analysis, clearly indicate that for both duplex steels the austenite phase shows a higher hardness due to work hardening induced by deep rolling as the ferrite phase.

**Figure 6:**
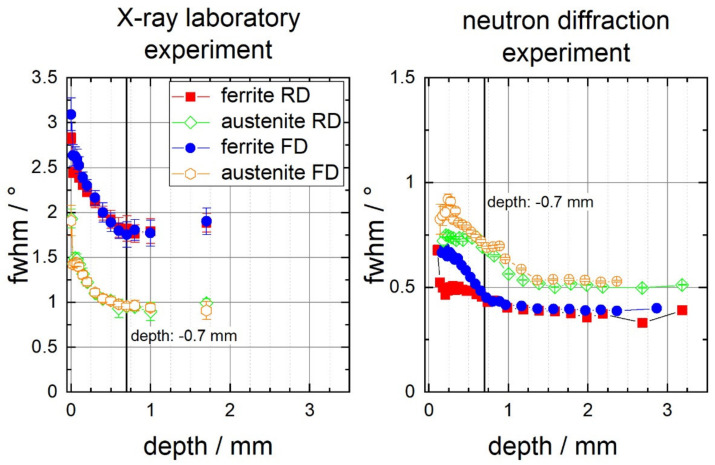
FWHM values of the ferrite and austenite interference lines of deep rolled duplex steel 1.4462 in the rolling direction (RD) and the feed direction (FD) determined by means of X-ray diffraction (**left**) and neutron diffraction (**right**). The data are based on the X-ray results.

Much clearer evidence for the depth range of the work hardening being induced by the deep rolling process can be gained from the phase-specific evolution of the full width at half maximum (FWHM) of the diffraction lines of austenite and ferrite, as determined by means of lab X-ray diffraction and neutron diffraction, respectively. As an example, for the duplex steel 1.4462, the depth distribution of the phase-specific mean FWHM values are plotted for the lab X-ray experiments for the rolling direction (RD) and the feed direction (FD) on the left hand side diagram, and the corresponding results for the neutron diffraction experiment are shown on the right hand side image of Figure 6. Since the peak width of the diffraction lines depends, among other factors, on the diffraction angle, the instrument broadening and lattice defects [37], the values depicted in Figure 6 differ depending on the {*hkl*} plane and the applied wavelength used for diffraction analysis. In the present laboratory measurement series using X-ray diffraction, the 2Θ line position for the unstressed lattice of the γ{220} line was around 128.3°, whereas the α{211} line was measured around 2Θ ≈ 156.4°. In contrast, in the neutron experiment, the γ{311} line is measured at a detector angle of about 103.5°, but the ferrite α{211} line is measured at 2Θ ≈ 91.5°. In consequence, the FWHM values for the unstressed lattice determined for the austenite {220} line is smaller than for the ferrite {211} line, but the FWHM value of austenite {311} line is larger than the ferrite {211} line determined in the neutron experiment. For one measurement series using identical measurement conditions, the FWHM values scale with the size of the coherently scattering regions and hence with the lattice imperfection density. In the current case, the change in FWHM values mainly scales with the dislocation density (see, e.g., [38,39,40]). In this regard, the increasing FWHM values in the near surface region correspond with the degree in work hardening induced by deep rolling. The higher depth resolution is achieved by means of X-ray diffraction analysis due to the small step-sizes for layer removal applied. Here, the depth distribution indicates that a depth of up to about 0.7 mm is affected by the deep rolling procedure, i.e., up to this depth, an increase in work hardening can be verified.

### 4.2. Texture and Texture Evolution 

**Figure 7:**
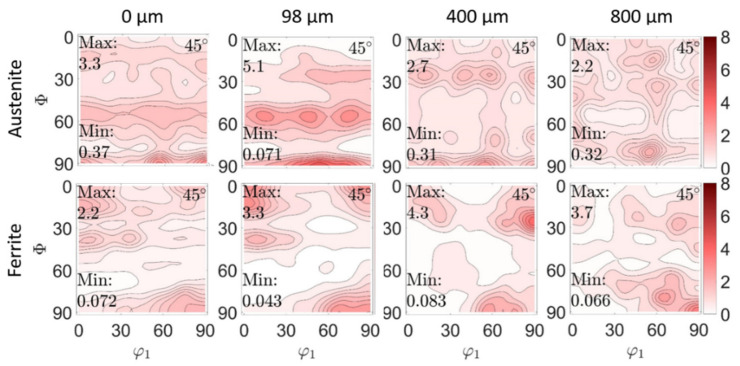
Crystallographic texture of the austenite and ferrite phases for the duplex stainless steel 1.4462, and evolution of the phase-specific texture in depth for the work hardened state (after deep rolling). The presented selected depths correspond with different degrees of work hardening. The initial texture is displayed in Figure 3. The color code on the right corresponds to the texture sharpness (m.r.d).

**Figure 8:**
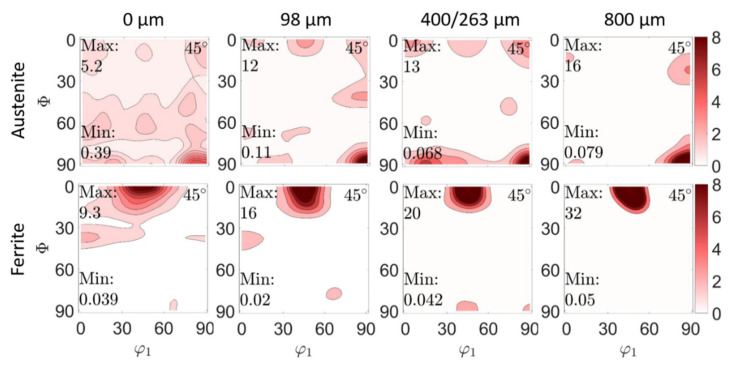
Crystallographic texture of the austenite and ferrite phases for the duplex stainless steel 1.4460, and evolution of the phase-specific texture in depth for the work hardened state (after deep rolling). The presented selected depths correspond with different degrees of work hardening. The initial texture is displayed in Figure 3.

In Figure 7 and Figure 8, results of phase-specific crystallographic texture analyses on both duplex steels, 1.4462 and 1.4460, are shown. The *φ*_2_ = 45° projections through the ODF of both phases, austenite and ferrite, are plotted for the material state after deep rolling for different depths. This was conducted to illustrate the texture evolution in both phases (ferrite and austenite) due to the gradient in plastic deformation that follows deep rolling. The figures clearly show that the initial texture of both duplex steels has been changed, with plastic deformation near the surface for both steel types investigated here. 

Phase-specific crystallographic textures for the initial state for both steels are shown in the previous chapter in Figure 3. At first, the in-depth distribution of the phase-specific texture is described for the duplex steel 1.4462 (Figure 7). Here, in the initial state in the ferrite phase, the maxima of the distribution at the φ_2_ = 45° cut are at (001)[1 −1 0], (001)[−1 −1 0] and at (75°,90°,45° with Eulerian angles φ_1_,Φ, φ_2_), which is close to the (110)[0 0 1] α-fibre [41,42]. Furthermore, there seems to be a slight {111} γ-fibre (at Φ = 55°) [43]. This {111} fiber is, together with the (110)[0 0 1] α-fibre, typical for the ferrite phase in rolled steels [42,43,44]. A pronounced γ-fiber may be desirable for some applications, as it positively influences the deep drawing properties [43]. The texture in the austenite phase is somewhat less pronounced (max. m.r.d. is 3.2 instead of 4.1). There also seems to be a slight γ-fibre (at Φ = 55°). The maximum appears at the Euler triple (55°, 90°, 45°), which could indicate a brass component [42]. After deep rolling at the very surface, no fundamental change of texture in the ferrite phase can be seen directly at the surface, but a significant weakening of the texture intensity (m.r.d. from 4.1 to 2.2) can be noticed. In the austenite phase, a constant superelevation (m.r.d. from 3.2 to 3.3) was measured. Here, the gamma fibre is sharper than in the initial state. The maximum characteristic of the γ-fibre is at about 100 µm depth. With increasing distance from the surface, i.e., with less amount of work hardening, a texture change is observed. At a depth of about 400 µm, the γ-fibre (austenite) disappears. Maxima form along Φ = 25° and 90°. At a depth of about 800 µm, a further change and the transition to the initial state (see Figure 3) is observed. In ferrite, a transition to the initial state is observed, starting at a depth of about 400 µm.

A comparable result of phase-specific texture and texture evolution, as a consequence of deep rolling, is presented for duplex stainless steel 1.4460 in Figure 8 (initial state in Figure 3c,d). Here, for the initial austenite phase, the maxima of the distribution were determined at the Eulerian angles (30°, 20°, 45°), (80°, 60°, 45°) and (30°, 50°, 45°) with the last two being along a possible γ-fibre. In the ferrite phase, the maximum is clear with a magnification factor (m.r.d.) of 29 at (0°, 45°, 45°). This is a very pronounced (001) [1 −1 0] texture, with other orientations being quasi-absent. This texture corresponds to a cube orientation [42]. After deep rolling at the very surface, the ferrite texture remains similar to the initial state, but indicates a very pronounced attenuation of the texture sharpness (m.r.d. from 29 to 9.3). At higher depth, the intensity of the distribution function increases again. From a depth of 800 µm, the initial texture (see Figure 3) is more or less restored. In the austenitic phase, a significant increase in intensity (m.r.d. from 2.5 to 5.2) is observed at the surface. A γ-fibre and a maximum at (90°, 90°, 45°), i.e., (110) [0 0 1] are also observed, which correspond to a Goss orientation [42]. This becomes more pronounced with increasing depth, whereas the γ-fibre dissipates. With increasing distance to the surface (max. at 800 µm), no transition to the initial state is observed in the austenite phase.

It is obvious that the two initial textures of the two duplex stainless steels, and their evolution, differ due to the deep rolling process. The different initial texture can be explained by the different process routes for the 1.4462 plate in comparison with the hot rolled 1.4460 bar. Furthermore, the observed weakening of the crystallographic texture, due to deep rolling, is accompanied with microstructural refinement (grain segmentation) in the region affected by work hardening induced by deep rolling. Together, these contribute to an improved determinability of RS by means of diffraction methods. 

Hence, regarding the γ{220}- and the α{211}-lattice planes, a much more homogeneous diffraction intensity distribution can be noticed for both materials in the two measured directions (rolling and feed direction) for the regions affected by the deep rolling compared to the initial state. Conversely, this means that without this homogenization effect through the deep rolling process, reliable X-ray stress analyses according to the *sin*^2^*ψ* method could not be carried out for the strongly textured initial state of both duplex stainless steels. Without weakening of the phase-specific texture sharpness, much more elaborate stress analysis approaches would have been necessary, such as, for instance, the crystallite group method described in [37].

### 4.3. Phase-Specific Residual Stress

**Figure 9:**
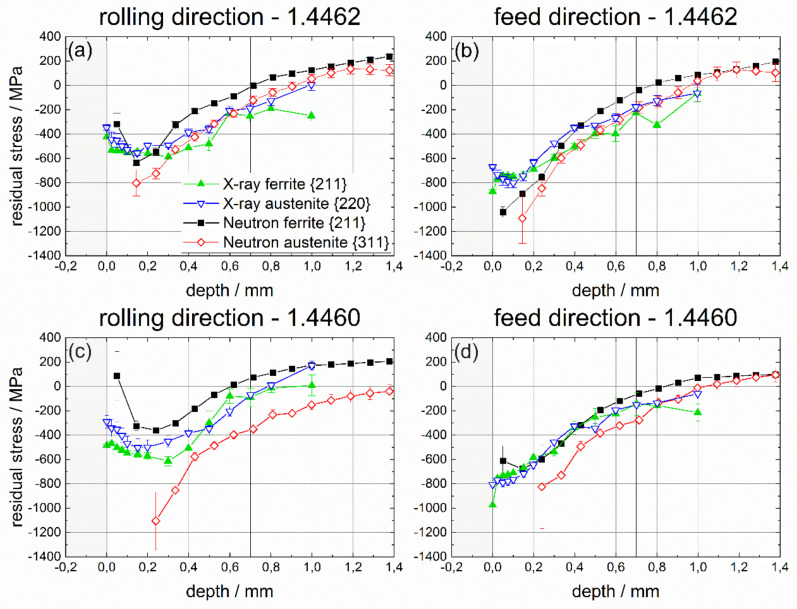
Phase-specific RS for ferrite and austenite determined by means of neutron diffraction and X-ray diffraction in both directions (in the rolling direction (DR) and in the feed direction (FD)) for both materials, i.e., for duplex steel 1.4462 with a phase ratio of 50:50 and for duplex steel 1.4460 with a ferrite to austenite ratio of 70:30.

**Figure 10:**
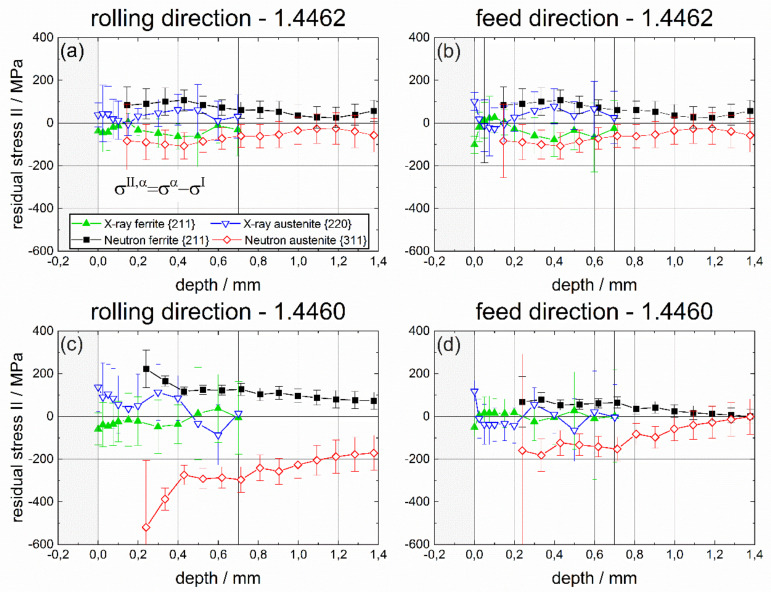
Phase-specific micro RS for ferrite and austenite determined by means of neutron diffraction and X-ray diffraction in both directions (in the rolling direction (DR) and in the feed direction (FD)) for both materials, i.e., for duplex steel 1.4462 with a phase ratio of 50:50 and for duplex steel 1.4460 with a ferrite to austenite ratio of 70:30.

**Figure 11:**
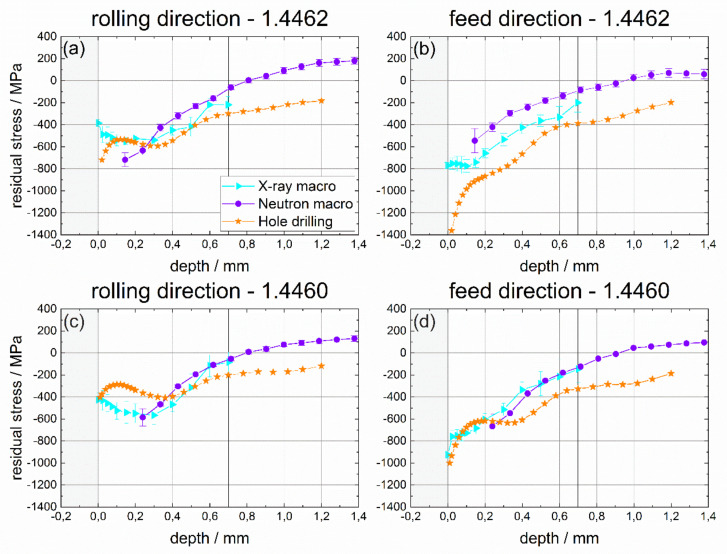
Macro RS determined by means of neutron diffraction, X-ray diffraction and incremental hole drilling in both directions (in the rolling direction (DR) and in the feed direction (FD)) for both materials, i.e., for duplex steel 1.4462 with a phase ratio of 50:50 and for duplex steel 1.4460 with a ferrite to austenite ratio of 70:30.

The non-destructive determination of phase-specific RS, after deep rolling of duplex stainless steels containing different austenite and ferrite phase fractions, was the focus of this project. In this regard, phase-specific neutronographic RS analyses were carried out using the instrument STRESS-SPEC at the research reactor FRM II at MLZ (Garching, Germany). The strain calculation was based on d_0_ reference values determined from ø 1.7 mm pins sectioned from the deep rolled samples. At first, a comparison of the results of non-destructive RS determination by means of neutron diffraction with results of X-ray stress analysis is presented. In Figure 9, the phase-specific RS are presented. In Figure 10, the phase-specific micro RS are shown, which are calculated using the respective phase fractions. Finally, in Figure 11 the macro RS are displayed that were calculated by subtracting the micro RS from the phase-specific ones. Here, the comparison with the complementary incremental hole drilling method is also considered. In all cases, the results for the rolling direction (RD) are presented in the partial diagrams (a) for the 1.4462 duplex steel and in (c) for the duplex steel 1.4460, respectively. The results for the feed direction (FD) are presented in partial images (b) for the material 1.4462 and in (d) for the duplex steel 1.4460. As a reminder, this means that for the interpretation of the results in the upper row, a phase ratio of 50:50 must be assumed, while, in the lower row, the ferrite to austenite ratio is 70:30.

As stated in the previous sub-chapter, one interesting result is that texture development induced by the work hardening enables meaningful X-ray stress analyses (XSA) according to the *sin*^2^*ψ-*method in the depth region affected by deep rolling, while, for the initial state of both duplex steels, XSA resulted in large errors due to the pronounced phase-specific crystallographic texture. In the initial state and, hence, also in larger depths, where the effect of work hardening decreases, no meaningful stress analysis by means of XSA was feasible. In consequence, we stopped XSA in a depth where the error was no longer justifiable. In other words, this means that, due to the work hardening induced by deep rolling, phase-specific texture plays a minor role for diffraction stress analysis, i.e., for measurement and for data evaluation.

For both duplex steels, the diffraction results presented in Figure 9 indicate that compressive phase-specific RS were induced by deep rolling with a maximum in compressive RS below the surface in a depth range between about 0.15 and 0.2 mm. Compressive RS are higher in the feed direction than in the rolling direction, which is in accordance with the literature [45,46,47] and can be explained by the surface pressure occurring in the contact zone between the deep rolling tool (ball) and the sample. During deep rolling, a triaxial stress state is always formed. This stress state is a direct consequence of the Hertzian pressure. The amounts of the resulting RS in the rolling and feed directions depend on the contact force and tool geometry. Plastic deformation and the resulting RS in the sample only occur if the equivalent stress resulting from the triaxial stress state is higher than the local yield strength of the material [47]. Furthermore, the results clearly show that, for both duplex steels, only a small amount of phase-specific micro RS is induced through the deep rolling process. This is obvious for the XSA results. Here, in both phases, almost identical phase-specific RS were determined. The neutron diffraction results show a good agreement with the XSA distributions. However, the difference between the austenite and the ferrite phase appears to be larger. In this regard, the *sin*^2^*ψ*-approach using X-ray diffraction is the more reliable procedure since reference values for the stress-free or stress-independent state have a minor impact on the RS data. In the backscatter range, even large deviations from the actual reference value will have low impact on the phase-specific RS, since it is only included in the cotangent and the RS are calculated on basis of relative shifts of the diffraction line positions, when tilting the sample about the distance angle ψ. However, for the lattice strain calculation using neutron diffraction, the reference value d_0_ was determined from a small pin by means of electro discharge machining (EDM), which directly affects the evaluation. Obviously, the phase-specific work hardening due to deep rolling induces small amounts of phase-specific micro RS that are not entirely relaxed after sectioning of the reference pin. This is in accordance with neutron diffraction studies on IN 718 [44] where it was discussed that the micro RS state in the reference pin is not entirely the same as in the parent IN 718 plate.

In consequence, in all cases, higher compressive RS were determined for the austenite phase, indicating that the austenite phase exhibits compressive micro RS, while, for the ferrite phase, tensile micro RS must exist. This is proven by the results depicted in Figure 10, where the phase-specific micro RS, calculated from the phase-specific RS under consideration of the existing phase fractions, are plotted for both duplex steels and both principal process directions, RD and FD. These micro RS are significantly lower for the duplex steel 1.4462 than for steel 1.4460 and are symmetrically distributed in both phases. The average micro RS of the XSA measurements are around ±50 MPa; the neutronographically determined values are higher and amount to about ±100 MPa on average. In duplex steel 1.4460, considerably higher micro RS occur in the austenite, which is mainly due to the lower volume content of austenite.

It is notable that the phase-specific micro RS based on X-ray diffraction (XSA) tend to be compressive in ferrite and tensile in the austenite phase. In contrast, neutronographic measurement results indicate a reversed sign for the phase-specific micro RS. Here, the ferrite phase exhibit tensile micro RS and the austenite phase compressive ones. In addition to the differing effect of the reference value on the determined RS, as stated above, it must be noted that there exists a fundamental difference in the RS analysis between X-ray diffraction and neutron diffraction. The strict application of the *sin*^2^*ψ*-method provides the deviatoric stress component only from the slope of the regression line 2θ vs. *sin*^2^*ψ*. The hydrostatic RS component results in the vertical shift of the regression line. However, these hydrostatic RS are generally rarely determined using this approach since this requires an accurate knowledge of the lattice parameters of the unstressed crystal lattice, which is problematic, particularly for coarse two-phase materials. In contrast, by means of neutron diffraction, the total stress tensor is determined for the principal directions, i.e., the sum of the hydrostatic and the deviatoric part and for coarse two-phase materials such as the duplex steels the hydrostatic stress tensor strongly depends on the reference value d_0_. This is problematic for the severely work hardened material states after deep rolling, since the sectioning does not result in an entire relaxation of the phase-specific micro RS.

Finally, the macro RS depth distributions are compared in Figure 11. For the assessment of the goodness of the macro RS depth distributions, determined using the two complementary diffraction approaches, additional RS analyses by means of incremental hole drilling were performed. Figure 11 shows the macro RS calculated from the phase-specific RS for the two principal directions, RD and FD, in comparison with the incremental hole drilling results. In accordance with the boundary conditions of incremental hole drilling (used rosette, hole diameter), the RS depth distributions were presented up to a depth of about 1.2 mm. However, it must be noted that the chosen evaluation approach (differential method), which is superior in the near surface region, tends to underestimate the residual stresses in larger depth. Hence, the data for depths >0.7 mm must be viewed critically.

The results determined using the three complementary approaches show very good agreement for the duplex steel 1.4462 in rolling direction (RD). The same holds for the feed direction (FD) in case of duplex steel 1.4460. The results of the neutron diffraction analysis fit perfectly to the macro RS determined by means of XSA and incremental hole drilling. Using the non-destructive approach, the macro RS distribution shows a region with compressive RS up to a depth range of about 0.8–0.9 mm. For larger depths, balancing tensile RS are determined. Principally, this also holds for duplex steel 1.4460 for the component in the rolling direction (FD). However, in the very near surface region, about 200 MPa lower compressive RS were determined by incremental hole drilling. For depths larger than 0.3 mm, excellent agreement between the mechanical and the two diffraction-based methods (neutron and X-ray diffraction) again exists. However, no reasonable explanation can be given for the deviating behaviour near the surface. The only RS distribution, where no satisfactory agreement between the mechanical method (hole drilling) with the diffraction approaches could be found, was the one displayed in diagram Figure 9b, i.e., for the duplex steel 1.4462 in feed direction (FD). Here, much higher compressive RS were determined close to the surface by means of incremental hole drilling in comparison to X-ray diffraction and neutron diffraction stress analysis. Close to the surface, values of up to about −1400 MPa were determined by the hole drilling method, while through XSA stresses were determined only around −800 MPa. However, this rather high overestimation of the compressive macro RS can be explained by the deficiency of the incremental hole drilling method to monitor high RS states correctly. Drilling a hole implies that a notch is introduced and, due to the notch effect, stress concentration occurs. For high RS, this results in localized plastic deformation in the vicinity of the drilled hole and, as a consequence, higher strain relaxations will be measured by means of the strain gauges. Since common stress calculation algorithms available for the incremental hole drilling method are based on linear elastic materials behaviour, erroneous RS are calculated. In the present case, the rather high compressive RS will be drastically overestimated. This is commonly termed the “plasticity effect”, which occurs for RS that exceed about 60–70% of the materials local yield stress [48].

## 5. Conclusions

In the present work, the phase-specific RS distributions of two coarse multiphase duplex steels were investigated. The experimental RS investigations were carried out using three different stress analysis methods. Here, the neutronographic RS measurement was supplemented by the laboratory X-ray $\sin^{2}\left(\psi \right)$ method and incremental hole drilling. Thus, a comprehensive analysis of phase-specific RS could be carried out. From the results, the following points could be shown within the scope of this work:

- The comparison of the macro RS distributions impressively shows that meaningful non-destructive analysis of near surface macro RS depth distributions, after deep rolling of coarse two phase materials such as duplex steels, can be carried out using neutron diffraction for depths starting at about 150–200 µm. For smaller depths, the scattered intensity is too low and the errors will not be justifiable. Therefore, close to the surface, complementary methods like X-ray stress analysis must be applied.
- It was shown that employing of neutron ray-tracing simulation to correct instrumental surface effects that usually occur in neutron through surface strain scanning is also applicable to multiphase materials and yields reliable results. Good agreements of residual stress distributions determined by using complementary analytical methods were obtained in this work.
- However, phase-specific micro RS determined by means of neutron diffraction show significant deviations to data determined by means of X-ray diffraction. This is due to the fact that the reference values determined by a small ø 1.7 mm pin sectioned from the deep rolled process zone clearly does not reflect the stress-free state. Phase-specific micro RS induced by work hardening were not entirely relaxed through the sectioning and, hence, significantly affect the lattice strain calculation.
- Furthermore, the investigations have shown that the pronounced texture of the initial state of both duplex steels was drastically changed by the deep rolling and the related work hardening. The phase-specific crystallographic texture was nearly distraught in the zone affected by work hardening. Consequently, the diffraction RS analysis by means of lab X-rays and neutrons were not affected by texture effects; hence, it was not necessary to consider crystallographic texture in the deep rolled materials region. This holds for the measurement as well as the subsequent evaluation of the diffraction data.
- Reliable diffraction stress analyses for the coarse two phase materials studied would not have been possible without the texture modification/attenuation caused by the deep rolling.

## Figures and Tables

**Table 1:** Chemical composition of duplex steels X2CrNiMoN22-5-3 (1.4462) [24] and X3CrNiMoN27-5-2 (1.4460) [25].

	C	C	Si	Mn	P	S	Cr	Mo	Ni	N
1.4462	0–0.03	0–0.03	0–1.0	0–2.0	0–0.035	0–0.015	21.0–23.0	2.5–3.5	4.5–6.5	0.1–0.22
1.4460	0–0.05	0–0.05	0–1.0	0–2.0	0–0.035	0–0.03	25.0–28.0	1.3–2.0	4.5–6.5	0.05–0.20

**Table 2:** Nominal and measured phase fractions and strength values for duplex steels X2CrNiMoN22-5-3 (1.4462) and X3CrNiMoN27-5-2 (1.4460).

	Nominal Ferrite Content/%	Measured Ferrite Content/%	Nominal Austenite Content/%	Measured Austenite Content/%	Nominal R_p0.2_/MPa	Measured Yield Strength/MPa
1.4462	50	44 ± 4%	50	56 ± 4%	448 [24]	575
1.4460	70	71 ± 10%	30	29 ± 10%	460 [25]	630

**Table 3:** Diffraction elastic constants (DEC) calculated on the basis of single crystal data according to the indicated references. The measurement methods indicate whether the DECs were used for evaluation of neutron and/or X-ray diffraction data.

Measurement Methods	Phase	Youngs Modulus [MPa]	Poisson’s Ratio [-]	Reference
Neutron/X-ray	α{211}	219,911	0.280	[30]
Neutron	γ{311}	177,329	0.341	[29]
X-ray	γ{220}	212,758	0.310	[29]

## Data Availability

The data presented in this study are available on request from the corresponding author.
